# Physiological characterization using standardized submaximal treadmill responses in professional male soccer players

**DOI:** 10.3389/fspor.2026.1979307

**Published:** 2026-10-14

**Authors:** Ágnes Sziva, János Pórszász, Zsuzsanna Kívés, Zsolt Szelid

**Affiliations:** 1 Doctoral School of Health Sciences, Faculty of Health Sciences, University of Pécs, Pécs, Hungary; 2 The Lundquist Institute for Biomedical Innovation at Harbor-UCLA Medical Center, Torrance, CA, United States; 3 Faculty of Health Sciences, Institute for Health Insurance, University of Pécs, Pécs, Hungary; 4 Department of Sports Medicine and Digital Health, University of Győr, Győr, Hungary

**Keywords:** anaerobic threshold, blood lactate, exercise testing, physiological profiling, professional soccer, running performance, sports performance, treadmill test

## Abstract

**Objectives:**

Submaximal treadmill variables are increasingly used in professional soccer, but their practical interpretation remains limited. Therefore, we aimed to determine whether standardized submaximal treadmill-derived variables provide a more informative characterization of positional differences than maximal exercise variables and to examine their associations with maximal exercise performance.

**Methods:**

In this retrospective cross-sectional study, professional adult male soccer players (*n* = 206), including midfielders, defenders, and forwards performed an incremental treadmill test until exhaustion, with enhanced speed-dosing above 4 mmol·L⁻^1^ blood lactate (4L). Absolute and relative heart rate (HR) and speed variables at 4L and exhaustion, together with their associations were analysed during this incremental treadmill test.

**Results:**

Midfielders reached the highest 4L-related values, including speed (V_4L_: 15.6 **±** 1.3 km/h, *p* < 0.005 vs others), HR (HR_4L_: 177.2 **±** 9.0/min, *p* < 0.05 vs others), V_4L_ as % of maximal speed (V*_4L/Max_* ratio: 77.6 **±** 4.9%, *p* < 0.005 vs. forwards), and HR_4L_ as % of HR reserve (HR*_4L/Res_* ratio: 87.1 **±** 4.9%, *p* < 0.05 vs. others). Positional differences at maximal exercise were inconsistent. V_4L_ correlated moderately with HR_4L_ in all players (*r* = 0.364, *p* < 0.001), largely with HR*_4L/Res_* ratio (*r* = 0.609, *p* < 0.001) and while HR*_4L/Res_* ratio correlated very largely with V*_4L/Max_* ratio (*r* = 0.795, *p* < 0.001), without positional differences. Between 4L and exhaustion, correlations were larger for HR, than for speed, being very large for HR in all (*r* = 0.739, *p* < 0.001), while large in forwards (*r* = 0.661, *p* < 0.001), and for speed large in all (*r* = 0.575, *p* < 0.001), across playing positions. After adjustment for age, BMI, club, competitive division, testing phase, and testing year, positional effects remained significant for V_4L_ (*p* = 0.003) and HR_4L_ (*p* = 0.007), but not for V_Max_ (*p* = 0.077) or HR_Max_ (*p* = 0.147).

**Conclusions:**

Submaximal treadmill-derived variables differentiated playing positions more consistently than maximal exercise variables, while remaining strongly associated with maximal treadmill performance. These findings highlight 4L-related heart rate and running speed variables as informative submaximal markers for the physiological characterization of professional soccer players.

## 1 Introduction

Soccer is a dynamic, high-intensity team sport that places substantial aerobic and anaerobic demands on players (1). During a match, the majority of the energy is supplied by aerobic metabolism (2), mainly during low- and moderate-speed running, whereas repeated sprints (3) predominantly rely on anaerobic energy pathways. Consequently, well-developed aerobic fitness is essential for maintaining performance throughout the match. Match-running demands, including total distance covered and distances covered during high-intensity running and sprinting, have previously been shown to vary according to playing position (4–6). Associations between match-running performance and match outcome have also been reported, although the observed relationships varied according to playing position and running intensity (7).

Laboratory-based incremental treadmill testing is widely used in professional soccer to assess aerobic fitness under standardized conditions (8–10) and its results are considered as important reference for validating field-based test results (11). Moreover, straight-line running, the movement pattern of the treadmill test, was previously described as the most frequent action in goal situations (12), and straight-line running speed was shown to correlate with levels of performance (13). Besides the maximal exercise variables, treadmill tests provide several submaximal markers and therefore additional insights into the aerobic fitness of the players. Valuable submaximal markers include running speed at 4 mmol·L⁻^1^ blood lactate (4L), which repeatedly showed strong correlation with the level and the training status of soccer players (14, 15), more closely than maximal oxygen uptake alone (5, 16, 17). Likewise, heart rate reserve-based variables have been proposed as practical indicators of physiological internal strain (18–20) and heart rate reserve has also been incorporated in a match-based internal load variable, the individual training impulse (21). In the present study, HR reserve-based variables were selected to account for individual differences between resting and maximal HR and to provide a standardized relative expression of cardiovascular strain, while recognizing that they represent one of several approaches to describing internal load, alongside other HR-based and subjective measures.

Previous research has examined position-specific lactate-based endurance profiles in professional soccer players, including running velocity at a fixed 4 mmol·L⁻^1^ blood lactate reference (22). However, these analyses have primarily focused on running velocity and lactate-based endurance parameters, while the relationship between external performance (running velocity) and internal physiological strain (heart rate) at the fixed 4 mmol·L⁻^1^ reference remains less well characterized. In particular, limited information is available on whether these external and internal responses, and their relationships with corresponding maximal responses, differ according to playing position. Accordingly, the present study focused specifically on outfield players, as goalkeepers have previously been shown to exhibit distinctly different endurance performance profiles (22). Maximal treadmill testing is time-consuming, therefore its integration into the demanding training schedules of professional teams often faces difficulties. Consequently, there is a growing need for standardized submaximal markers that provide meaningful physiological characterization without relying exclusively on maximal exercise responses. A fixed 4L blood lactate reference (11, 23) offers a standardized physiological benchmark that facilitates direct comparison between players and repeated assessments over time, making it potentially useful for routine comparisons in elite team sports.

Therefore, the aim of the present study was to characterize positional differences in standardized submaximal treadmill-derived physiological variables obtained at the fixed 4 mmol·L⁻^1^ blood lactate reference and to compare these differences with those observed for maximal exercise variables. We hypothesized that the submaximal variables would show clearer positional differences than maximal exercise variables and would be positively associated with the corresponding maximal exercise responses. We further hypothesized that treadmill-derived external performance (running velocity) would be positively associated with internal physiological strain (heart rate). Other analyses of relationships among relative variables and position-specific correlation patterns were considered exploratory.

## 2 Methods

### 2.1 Study design and participants

This retrospective cross-sectional study included professional (24) male outfield soccer players from four Hungarian teams competing in the Hungarian first and second divisions. All players were active members of their respective squads between 2018 and 2025 and underwent regular performance screening as part of their clubs’ medical programs. Although players were recruited over multiple seasons and from four different clubs, all assessments were conducted by the first author of this manuscript and her colleagues, using the same standardized protocol. Treadmill testing was conducted semi-annually, during the early phase of team conditioning periods, following summer breaks (preseason) and winter breaks (midseason). For players with repeated assessments, only the first eligible test was included in the present analysis, irrespective of whether it was performed during the summer or winter preparation period. Goalkeepers were excluded from the study due to the previously described major difference in running capacities (5, 16, 25). Consequently, data from 206 asymptomatic, healthy outfield players were included in the final analysis, who completed the maximal effort treadmill test. Playing position at the time of the included test was assigned according to the classification provided by the player's club. For players who played in more than one position, the position in which they played most frequently was used for classification.

The study was conducted in accordance with the Declaration of Helsinki. Ethical approval for the retrospective analysis of the routinely collected data was obtained from the Hungarian Medical Research Council Scientific and Research Ethics Committee (ETT TUKEB, BM/17634-3/2023) and all data were processed anonymously in compliance with data protection regulations.

### 2.2 Exercise testing protocol

All players had previous experience with treadmill running; therefore, no separate familiarization session was performed. Before testing, the protocol and the procedure for safely terminating the test were explained to each player. Players had performed no training during the preceding 48 h. All tests were conducted in the same laboratory setting using a motorized treadmill ergometer (HP Cosmos Quasar med, 170–190/65). A two-phase treadmill protocol was used for routine performance testing in this study. Before testing, players remained seated at rest for at least 5 min without preceding exercise and baseline measurements were performed, including heart rate and capillary blood lactate determination. Baseline heart rate (HR_Base_) was recorded as a single heart rate value provided by the automated blood pressure monitor during the subsequent resting blood pressure measurement. In the first phase players performed a custom incremental ramp protocol adapted to the physiological demands of elite soccer. This began with a 2-minute warm-up at 5 km·h⁻^1^, followed by a 2-minute warm-up at 10 km·h⁻^1^, which also served as the initial stage of the protocol. Subsequently, treadmill speed was increased by 1 km·h⁻^1^ every 2 min, with 60 s breaks between each stage in standing position for capillary blood sampling, until capillary blood measurement exceeded the lactate value of 4 mmol·L⁻^1^ (4L). A fixed blood lactate concentration of 4 mmol·L⁻^1^ has traditionally been used to identify the onset of blood lactate accumulation (OBLA) and as a standardized reference related to the second lactate-threshold domain (26, 27). Beyond this point during the second phase of the test, treadmill speed was increased 1 km·h⁻^1^ every minute without breaks, to allow high-speed running until voluntary exhaustion. The treadmill grade was fixed at 0% throughout the test. After exhaustion, players were immediately seated and remained seated throughout a 5-minute recovery period, during which heart rate and ECG monitoring were continued and blood lactate was measured exactly 2 min after exercise cessation (La_2min_).

### 2.3 Performace and physiological variables: running speed, heart rate and lactate

The treadmill speed was adjusted as described before, up to the final speed at exhaustion (V_Max_). V_Max_ was defined as the highest treadmill speed maintained for at least 15 s; if the final speed was maintained for <15 s, the preceding speed was recorded as VMax. Test termination due to pain, injury, loss of running control, or another reason unrelated to volitional exhaustion was not considered a valid maximal test. The absolute running speed corresponding to the 4 mmol·L⁻^1^ blood lactate (V_4L_) was calculated by linear interpolation between the two consecutive exercise stages surrounding the 4L threshold value. When blood lactate concentration was below 4L at one stage and above 4L at the subsequent stage, the exact V_4L_ was estimated using the two-point equation of a straight line, as described before (14). V_4L_ was also expressed as percentage of the maximal speed (V*_4L/Max_* ratio).

Heart rate data were collected continuously throughout the entire test using a Polar H7 Heart Rate Sensor and recorded via a Polar M400 GPS running watch (Polar Electro Oy, Kempele, Finland). Data were synchronized with the Polar Flow web service and exported in CSV format as time-stamped heart-rate values recorded at 1-s intervals for further analysis. No temporal averaging or smoothing filters were applied. The following heart rate values were obtained from the recordings: baseline heart rate following at least 5 min of seated rest (HR_Base_), heart rate at the 4 mmol·L⁻^1^ blood lactate reference point (HR_4L_), and maximum heart rate (HR_Max_). HR_Max_ was defined as the highest physiologically plausible heart-rate value in the exported 1-s time series recorded during the test. Isolated implausible values attributable to signal artifacts were disregarded; in cases of suspected signal interference, heart rate was verified against the simultaneously recorded 12-lead exercise ECG. Continuous ECG monitoring was performed using a COSMED Stress PC ECG system (COSMED, Rome, Italy; Stress PC ECG Application, version 5.515961). The same heart-rate measurement and data-processing procedures were used throughout the study period.

The HR_4L_ was calculated on the basis of the previously calculated 4L speed (V_4L_) using straight line parallelism (14). Heart rate reserve (HR_Res_) was determined as the difference between the HR_Max_ and the HR_Base_ and percentage of the HR_Res_ (%HR_Res_) was calculated for heart rate at the 4L threshold (HR*_4L/Res_* ratio) as follows: HR*_4L/Res_* ratio* = ((HR_4L—_HR_Base_)/HR_Res_*) * 100. The heart rate at the 80% of the heart rate reserve (80%HR_Res_) was also determined and the corresponding speed (V80%HR_Res_) was calculated.

For blood lactate measurement, capillary blood samples were obtained from the fingertip by healthcare-qualified personnel experienced in capillary blood sampling. Before each sample, sweat and potential surface contamination were removed with a dry swab; the site was then disinfected and allowed to dry completely before puncture. The first drop of blood was discarded, and a subsequent drop was used for analysis. Fingers were alternated when appropriate to minimize discomfort and avoid repeated puncture of the same site. Blood sampling was performed before the test (La_Base_), at the end of each stage of the incremental treadmill protocol until exceeding 4 mmol·L⁻^1^ lactate value, and 2 min after completion of the test in a seated position (La**_2min_**), to detect the training-related delayed elevation of the capillary blood lactate (28), therefore resulting in a minimum of 7 lactate samples per participant. Blood lactate concentration was measured using a handheld analyser (Lactate Scout 4, SensLab GmbH, Leipzig, Germany). The fixed blood lactate concentration of 4 mmol·L⁻^1^ was used to define the onset of blood lactate accumulation (OBLA) (27).

### 2.4 Statistical analysis

Descriptive statistics included means and standard deviations (SD). Normality of data distribution was assessed using the Shapiro–Wilk test. Associations between treadmill-derived velocity, heart rate and blood lactate variables were examined using Pearson's correlation coefficient for normally distributed variables with linear relationships. For variables that did not meet assumptions of normality, Spearman's rank correlation was applied. Pearson correlation coefficients are reported as ***r*** and Spearman's rank correlation coefficients as ***ρ***. Correlation coefficients were used as suggested previously, with thresholds of 0.1 for small, 0.3 for moderate, 0.5 for large, 0.7 for very large and 0.9 for extremely large (29). For each correlation analysis, the correlation coefficient (***r*** or ***ρ***, as appropriate), *p*-value, sample size, and 95% confidence interval were reported. To address potential mathematical coupling between the normalized indices, additional sensitivity analyses were performed using the underlying non-normalized variables. Spearman's rank correlations were used because some variables deviated from normality. Multiple linear regression analyses were subsequently performed to examine the association between V_4L_ and HR_4L_ while accounting for the corresponding components used in normalization. To examine the discriminatory information provided by submaximal and maximal treadmill variables with respect to playing position, multinomial logistic regression analyses were performed with playing position (DEF, MID, FW) as the dependent variable. Separate models included either the submaximal variables V_4L_ and HR_4L_ or the maximal variables V_Max_ and HR_Max_ as predictors. A combined model including all four variables was additionally fitted. Model performance was evaluated using the likelihood-ratio test, Nagelkerke pseudo-R^2^, and overall classification accuracy. Group differences were evaluated using one-way analysis of variance (ANOVA) for normally distributed data or the Kruskal–Wallis test for non-parametric data. When significant overall effect was observed, *post hoc* comparisons were performed using Tukey's honestly significant difference (HSD) test following ANOVA or Mann–Whitney U test with Bonferroni correction following Kruskal–Wallis analyses, as appropriate. Effect sizes for overall positional comparisons were reported as eta squared (*η*^2^) for ANOVA and epsilon squared (*ε*^2^) for Kruskal–Wallis tests, with bootstrap 95% confidence intervals. For significant non-parametric pairwise comparisons, Hodges–Lehmann estimates with 95% confidence intervals were additionally reported. To assess whether positional differences were independent of potential confounding factors, additional general linear models were performed for the principal submaximal (V_4L_ and HR_4L_) and maximal (V_Max_ and HR_Max_) treadmill variables. Playing position, club, competitive division, testing phase (pre-season or mid-season), and testing year were entered as fixed factors, while age and body mass index (BMI) were included as covariates. Main effects were evaluated using Type III sums of squares, and effect sizes were reported as partial eta squared (*η*p^2^). When a significant main effect of playing position was observed, Bonferroni-adjusted pairwise comparisons of estimated marginal means were performed. Because club and competitive division were strongly associated, sensitivity models were also fitted including either club or competitive division separately. Statistical significance was accepted at *p* < 0.05 (two-tailed). Data were entered into Microsoft Excel for Microsoft 365 MSO version 2606 (Microsoft Corp., Redmond, WA, USA) and analysed using IBM SPSS Statistics version 29.0.2.0 (IBM Corp., Armonk, NY, USA). Figures were prepared using SigmaPlot version 16.0.0.28 (Grafiti LCC, Palo Alto, CA, USA). Because this was a retrospective analysis, no *a priori* sample size calculation was performed; all eligible players meeting the inclusion criteria were included.

## 3 Results

Data from 206 professional male outfield players (mean age: 23.3 ± 5.0 years) from four clubs competing in the first and second Hungarian soccer divisions were analysed. Complete data were available for all 206 players for all variables included in the analyses, with no outcome-specific missing data. When stratified by playing position, the sample was distributed evenly among defenders (DEF, *n* = 66; 32%), midfielders (MID, *n* = 74; 36%), and forwards (FW, *n* = 66; 32%). Age distributions were not different between the playing positions, with the following values in DEF, MID and FW players: 23.9 ± 5.0, 23.3 ± 4.8 and 22.7 ± 5.4, respectively (*p* = 0.379). Body mass index was similarly not different between positions (in DEF: 23.2 ± 1.5, in MID: 22.9 ± 1.3, in FW: 23.1 ± 1.3; *p* = 0,466).

### 3.1 Speed values and heart rates in different positions

Running speed varied significantly by playing positions, with more pronounced differences at the 4L threshold, than at maximal exertion. Detailed descriptive statistics, effect sizes, confidence intervals and *post hoc* comparisons are presented in Table 1. V_4L_ was highest in the midfielder group compared with both other outfield positions, whereas V_Max_ did not differ significantly between playing positions (*p* = 0.056). Although an overall positional difference was observed for V80%HR_Res_, no pairwise comparison remained significant after Tukey adjustment. Positional differences in absolute speed-related variables were accompanied by a higher V*_4L/Max_* ratio in midfielders compared with forwards. For heart rate, baseline absolute values did not differ significantly between playing positions. During exercise, however, positional differences were observed, with the highest HR_4L_ in midfielders, compared to both other outfield player groups, while at peak exercise, the HR_Max_ differences between positions were again less consistent, with higher value in midfielders, compared to defenders, but not versus forward players. When the HR_4L_ was expressed as percentage of the HR_Res_ (HR*_4L/Res_* ratio), it was again the highest in midfielders, compared to both other groups. Capillary blood lactate concentration measured 2 min after exercise cessation (La_2min_) was 12.0 ± 2.9 (mean ± STD) following the test, with no difference between playing positions (*p* = 0.903).

**Table 1:** Treadmill-based speed, heart rate and lactate variables in professional male soccer players in different playing positions (*N* = 206).

Variables	All (*n* = 206)	Defenders (*n* = 66)	Midfielders (*n* = 74)	Forwards (*n* = 66)	Total *p*-value	Effect size (95% CI)	Post hoc comparison, adjusted *p*-value and HL difference (95% CI)
HR_Base_ (bpm)	69.7 ± 13.2	70.5 ± 14.8	69.2 ± 12.2	69.3 ± 12.8	0.955^b^	*ε*^2^ = 0.000 (0.000–0.030)	—
HR_4L_ (bpm)	174.4 ± 10.0	172.5 ± 10.2	177.2 ± 9.0	173.3 ± 10.2	0.006^b^	ε^2^ = 0.040 (0.002–0.120)	DEF vs. MID: *p* = 0.009, −4.78 (−8.11 to −1.77) bpm; MID vs. FW: *p* = 0.048, 4.00 (0.81 to 6.94) bpm
HR_Max_ (bpm)	191.9 ± 8.7	190.3 ± 9.0	193.2 ± 7.9	191.9 ± 9.0	0.040^b^	ε^2^ = 0.022 (0.000–0.090)	DEF vs. MID: *p* = 0.033, −4.00 (−6.00 to −1.00) bpm
Percentage of HR_Res_ at 4L (%)	85.7 ± 5.5	85.0 ± 5.2	87.1 ± 4.9	84.8 ± 6.1	0.010^b^	ε^2^=.035 (0.000–0.120)	DEF vs. MID: *p* = 0.019, −1.99 (−3.40 to −0.59) percentage points; MID vs. FW: *p* = 0.044, 2.04 (0.40 to 3.61) percentage points
HR at 80% of HR_Res_ (bpm)	167.4 ± 8.3	166.3 ± 9.0	168.4 ± 7.6	167.4 ± 8.4	0.119^b^	ε^2^ = 0.011 (0.000–0.070)	—
V_4L_ (km·h⁻^1^)	15.2 ± 1.4	15.0 ± 1.5	15.6 ± 1.3	14.8 ± 1.4	<0.001^b^	ε^2^ = 0.075 (0.020–0.170)	DEF vs. MID: *p* = 0.009, −0.69 (−1.10 to −0.22) km·h^−^^1^; MID vs. FW: *p*<0.001, 0.91 (0.50 to 1.31) km·h^−^^1^
V_Max_ (km·h⁻^1^)	19.9 ± 1.3	19.8 ± 1.2	20.2 ± 1.1	19.7 ± 1.4	0.056^b^	ε^2^ = 0.019 (0.000–0.090)	—
V_4L_ as percentage of V_Max_ (%)	76.3 ± 5.8	76.0 ± 6.2	77.6 ± 4.9	75.1 ± 6.1	0.017^b^	ε^2^ = 0.031 (0.000–0.110)	MID vs. FW: *p* = 0.013, 2.36 (0.70 to 3.80) percentage points
V80%HR_Res_ (km·h⁻^1^)	13.8 ± 1.2	13.8 ± 1.3	14.0 ± 1.1	13.5 ± 1.3	0.046^a^	*η*^2^ = 0.024 (0.002–0.088)	—
La_2min_ (mmol·L⁻^1^)	12.0 ± 2.9	12.1 ± 3.3	11.7 ± 2.4	12.1 ± 3.0	0.903^b^	ε^2^ = 0.000 (0.000–0.030)	—

Data are presented as mean ± SD. ᵃOne-way ANOVA; ᵇKruskal–Wallis test. Effect sizes are reported as eta-squared (η^2^) for one-way ANOVA and epsilon-squared (ε^2^) for Kruskal–Wallis tests, with bootstrap 95% confidence intervals. Pairwise *post hoc* comparisons following significant Kruskal–Wallis tests were performed using Mann–Whitney U tests with Bonferroni adjustment; Hodges–Lehmann (HL) estimates and 95% confidence intervals are reported for significant pairwise comparisons. Tukey's test was used for *post hoc* comparisons following one-way ANOVA.

HRBase, heart rate before the test; HR4L, heart rate at the fixed blood lactate concentration of 4 mmol·L*^−1^*; HR_Max_, maximum heart rate; HRRes, heart rate reserve; V4L, running speed at the fixed blood lactate concentration of 4 mmol·L*^−1^*; Vmax, maximum running speed; V80%HRRes, running speed at 80% of heart rate reserve; La_2min_, blood lactate concentration at 2 min after exercise cessation; DEF, defenders; MID, midfielders; FW, forwards.

After adjustment for age, BMI, club, competitive division, testing phase, and testing year, playing position remained significantly associated with V_4L_ and HR_4L_. Adjusted pairwise comparisons showed higher V_4L_ in midfielders than in forwards, while HR_4L_ was higher in midfielders than in both defenders and forwards. In contrast, no significant positional effect was observed for V_Max_ or HR_Max_ (Table 2).

**Table 2:** Adjusted associations between playing position and submaximal and maximal treadmill-derived variables.

Outcome	Position F (df)	*p*	Partial η^2^	Significant Bonferroni comparisons	Adjusted mean difference (95% CI)
V_4L_	6.158 (2,188)	0.003	0.061	MID vs. FW	0.828 (0.254–1.402) km·h⁻^1^
HR_4L_	5.044 (2,188)	0.007	0.051	MID vs. DEF	4.366 (0.465–8.266) bpm
				MID vs. FW	4.499 (0.555–8.443) bpm
V_Max_	2.602 (2,188)	0.077	0.027	None	—
HR_Max_	1.935 (2,188)	0.147	0.020	None	—

General linear models were adjusted for age, BMI, club, competitive division, testing phase, and testing year. Playing position, club, competitive division, testing phase, and testing year were entered as fixed factors; age and BMI were included as covariates. Pairwise comparisons are Bonferroni-adjusted. Only statistically significant pairwise comparisons are shown.

### 3.2 Correlations between speeds, heart rates and lactate

Treadmill speed, heart rates and lactate levels were correlated during the test, using absolute and relative values. For all players a moderate correlation was present at the 4L between HR_4L_ and V_4L_ values (*r* = 0.364; *p* < 0.001; 95%CI: 0.239-0.477). When this correlation was determined in different positions, in forwards a similarly moderate correlation was observed (*r* = 0.422; *p* < 0.001; 95%CI: 0.202-0.603), but for the other groups this correlation was only small (for defenders *r* = 0.299; *p* = 0.015; 95%CI: 0.061-0.505 and for midfielders *r* = 0.268; *p* = 0.021; 95%CI: 0.042-0.467). However, when the percent of heart rate reserve at 4L (HR*_4L/Res_* ratio) was correlated with the V_4L_, a large correlation was observed for the entire group of players (*r* = 0.609; *p* < 0.001; 95%CI: 0.515-0.688) and this correlation was similarly large in defenders (*r* = 0.613; *p* < 0.001; 95%CI: 0.436-0.745), in midfielders (*r* = 0.575; *p* < 0.001; 95%CI: 0.399-0.710), and in forwards (*r* = 0.586; *p* < 0.001; 95%CI: 0.400-0.725).

The HR*_4L/Res_* ratio was also correlated with V*_4L/Max_* ratio, which revealed a very large correlation for the entire cohort (*r* = 0.795; *p* < 0.001; 95%CI: 0.739-0.841) (Figure 1). This correlation remained very large across all outfield positions, including in FW (*r* = 0.833; *p* < 0.001; 95%CI: 0.741-0.895), in DEF (*r* = 0.790; *p* < 0.001; 95%CI: 0.677-0.866) and MID (*r* = 0.738; *p* < 0.001; 95%CI: 0.613-0.827) players (Figure 2). La2 min showed inverse correlations with relative heart rates and speed values at the 4L, the HR*_4L/Res_* ratio and the V*_4L/Max_* ratio. A moderate correlation was observed between La_2min_ and HR*_4L/Res_* ratio in the total cohort (*r* = −0.496; *p* < 0.001; 95%CI: −0.592- −0.385), with similarly moderate correlations in defenders (*r* = −0.452; *p* < 0.001; 95%CI: −0.625- −0.235) and in midfielders (*r* = −0.498; *p* < 0.001; 95%CI: −0.653- −0.305), while in FW players a large correlation was observed (*r* = −0.536; *p* < 0.001; 95%CI: −0.689- −0.338). When La_2min_ was correlated with V*_4L/Max_* ratio, a large correlation was present in all players (*r* = −0.581; *p* < 0.001; 95%CI: −0.665- −0.483), but this negative correlation was different between positions, being large in FW (*r* = −0.689; *p* < 0.001; 95%CI: −0.798- −0.537) and in MID (*r* = −0.578; *p* < 0.001; 95%CI: −0.713- −0.403) players, while moderate in defenders (*r* = −0.486; *p* < 0.001; 95%CI: −0.651- −0.276).

**Figure 1:**
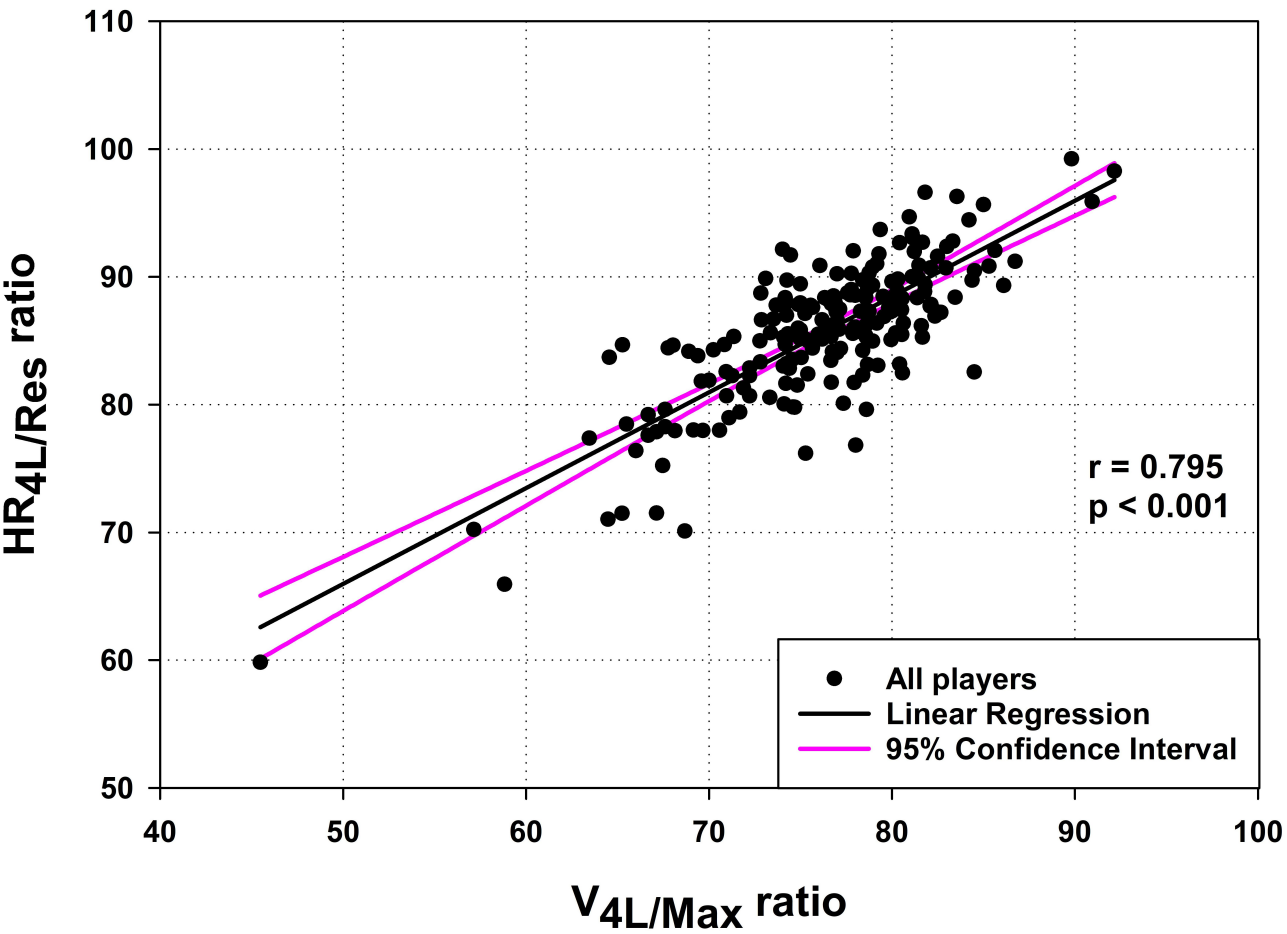
Correlations between treadmill-based percentage of the HR_Res_ at the 4L threshold (HR*_4L/res_* ratio) and percentage of the speed at 4L of the maximal speed (V*_4L/max_* ratio) in professional adult soccer players (*n* = 206). 95%CI: 0.739-0.841.

**Figure 2:**
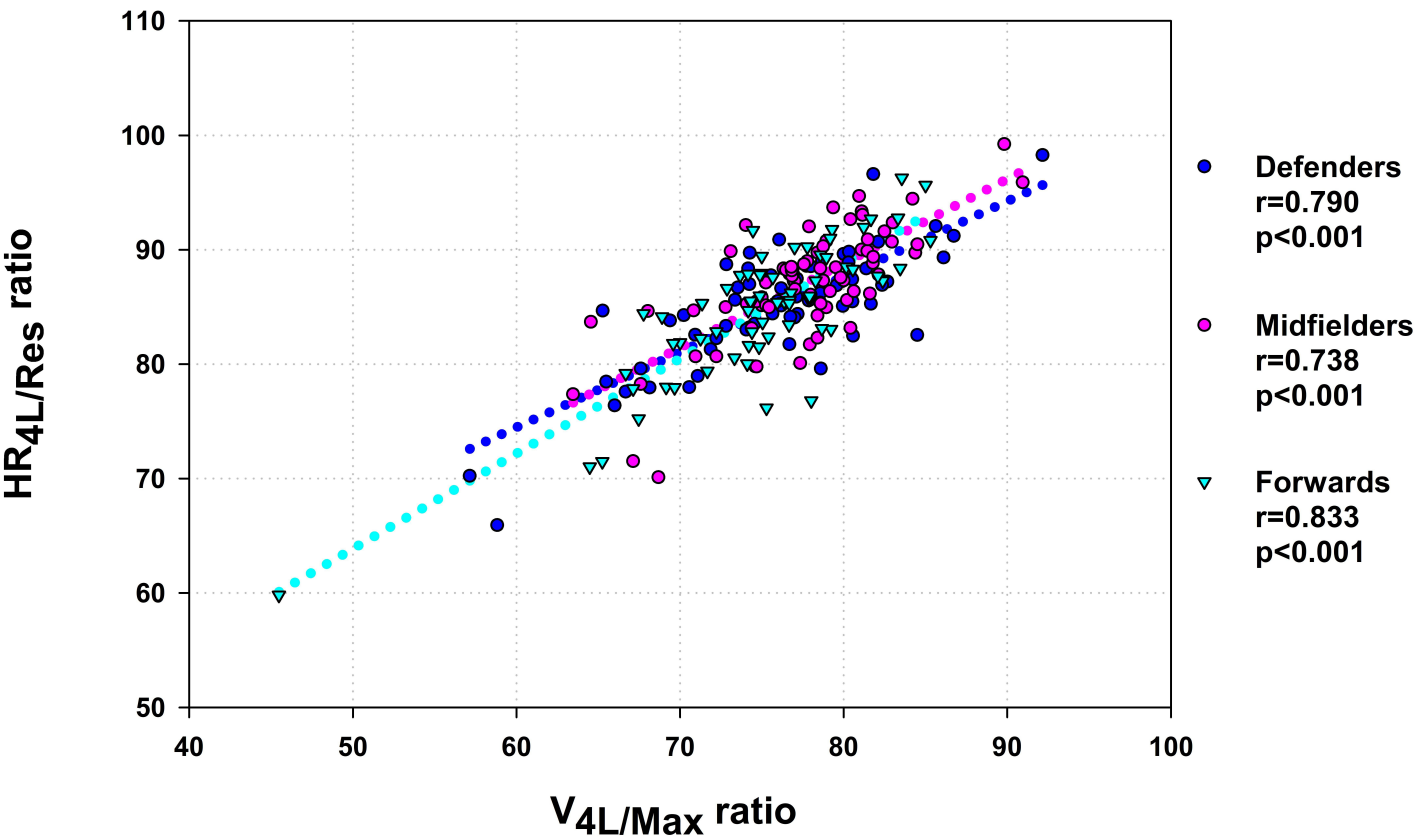
Correlations between treadmill-based percentage of the HR_Res_ at the 4L threshold (HR*_4L/res_* ratio) and percentage of the speed at 4L of the maximal speed (V*_4L/max_* ratio) in professional adult soccer players in different positions. 95% CI in DEF: 0.677-0.866, in MID: 0.613-0.827, in FW: 0.741-0.895. DEF: defenders; MID: midfielders; FW: forwards.

### 3.3 Correlation between variables at submaximal and maximal level

The correlation between V_4L_ and V_Max_ was large in the total sample (*r* = 0.575; *p* < 0.001; 95%CI: 0.475-0.660; Figure 3), and was similarly large in the MID group (*r* = 0.615; *p* < 0.001; 95%CI: 0.450-0.740), in the DEF group (*r* = 0.567; *p* < 0.001; 95%CI: 0.377-0.712) and in the FW group (*r* = 0.513; *p* < 0.001; 95%CI: 0.310-0.672) of players (Figure 4). Speed corresponding to heart rate at 80% heart rate reserve (V80%HR_Res_) showed a large positive correlation with maximal running speed (V_Max_) in all players (*r* = 0.664; *p* < 0.001; 95%CI: 0.580-0.734), with a very large correlation in defenders (*r* = 0.738; *p* < 0.001; 95%CI: 0.604-0.831), and large correlation in midfielders (*r* = 0.645; *p* < 0.001; 95%CI: 0.488-0.761) and in forwards (*r* = 0.602; *p* < 0.001; 95%CI: 0.422-0.737).

**Figure 3:**
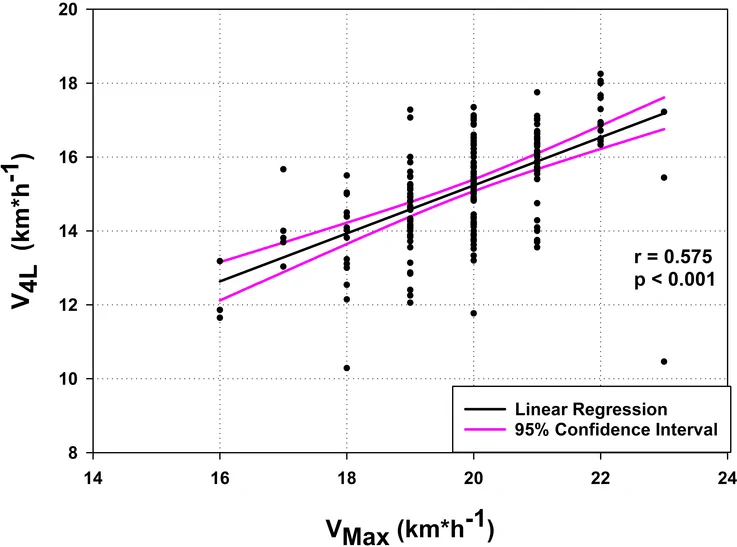
Correlations between treadmill-based speed at the 4L threshold (V_4L_) and maximal speed (V_Max_) in professional adult soccer players (*n* = 206). 95%CI: 0.475-0.660.

**Figure 4:**
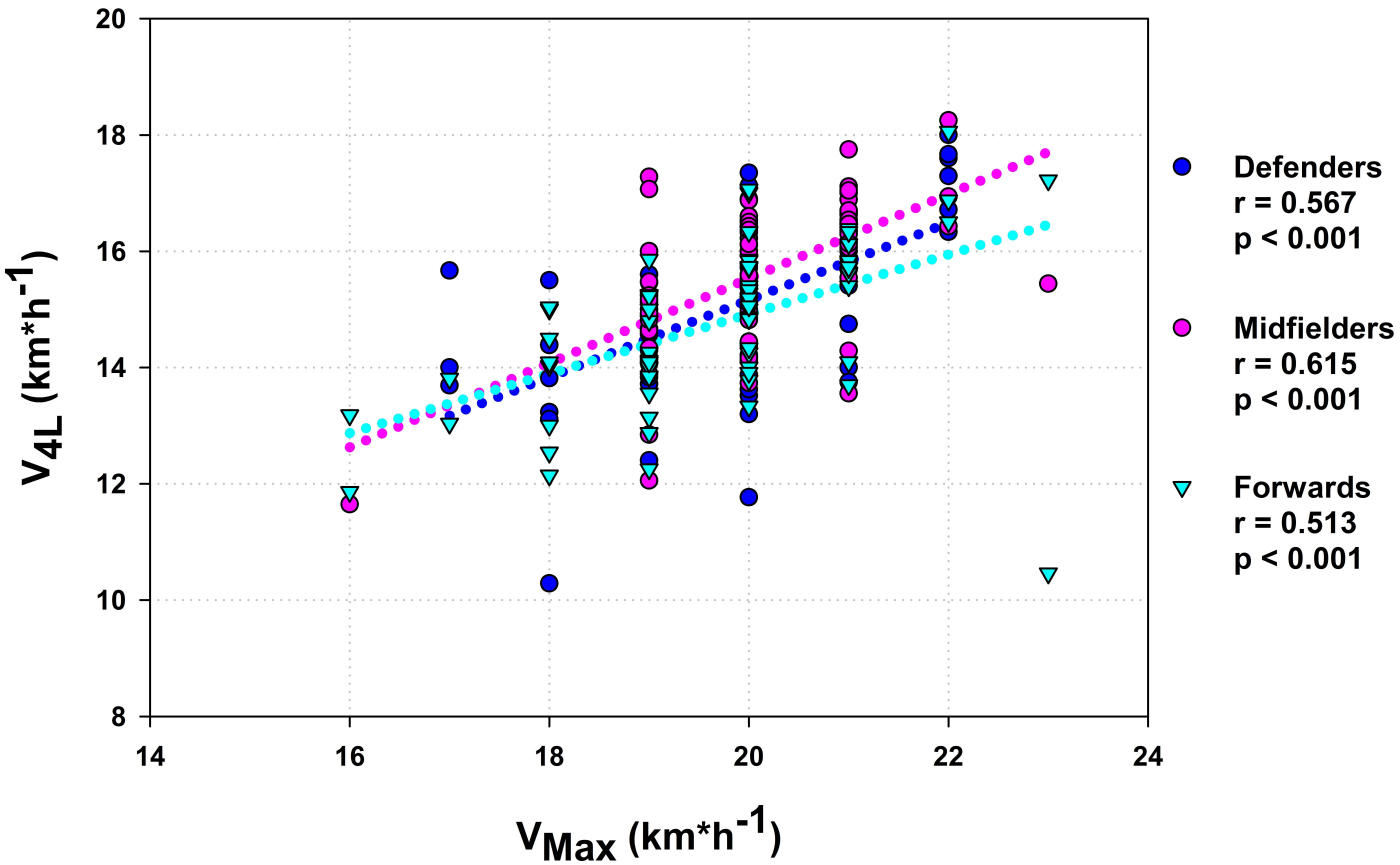
Correlations between treadmill-based speed at the 4L threshold (V_4L_) and maximal speed (V_Max_) in professional adult soccer players in different positions. 95%CI in DEF: 0.377-0.712, in MID: 0.450-0.740, in FW: 0.310-0.672. DEF: defenders; MID: midfielders; FW: forwards.

Absolute heart rate values at the 4L threshold (HR_4L_) showed very large correlation with the maximal heart-rate response (HR_Max_) in all players (Figure 5), *r* = 0.739; *p* < 0.001; 95%CI: 0.671-0.796), presenting a very large correlation in the defender group (*r* = 0.808; *p* < 0.001; 95%CI: 0.704-0.878; Figure 6) and in midfielders (*r* = 0.742; *p* < 0.001; 95%CI: 0.618-0.830), while large in forwards (*r* = 0.661; *p* < 0.001; 95%CI: 0.499-0.778; Figure 6).

**Figure 5:**
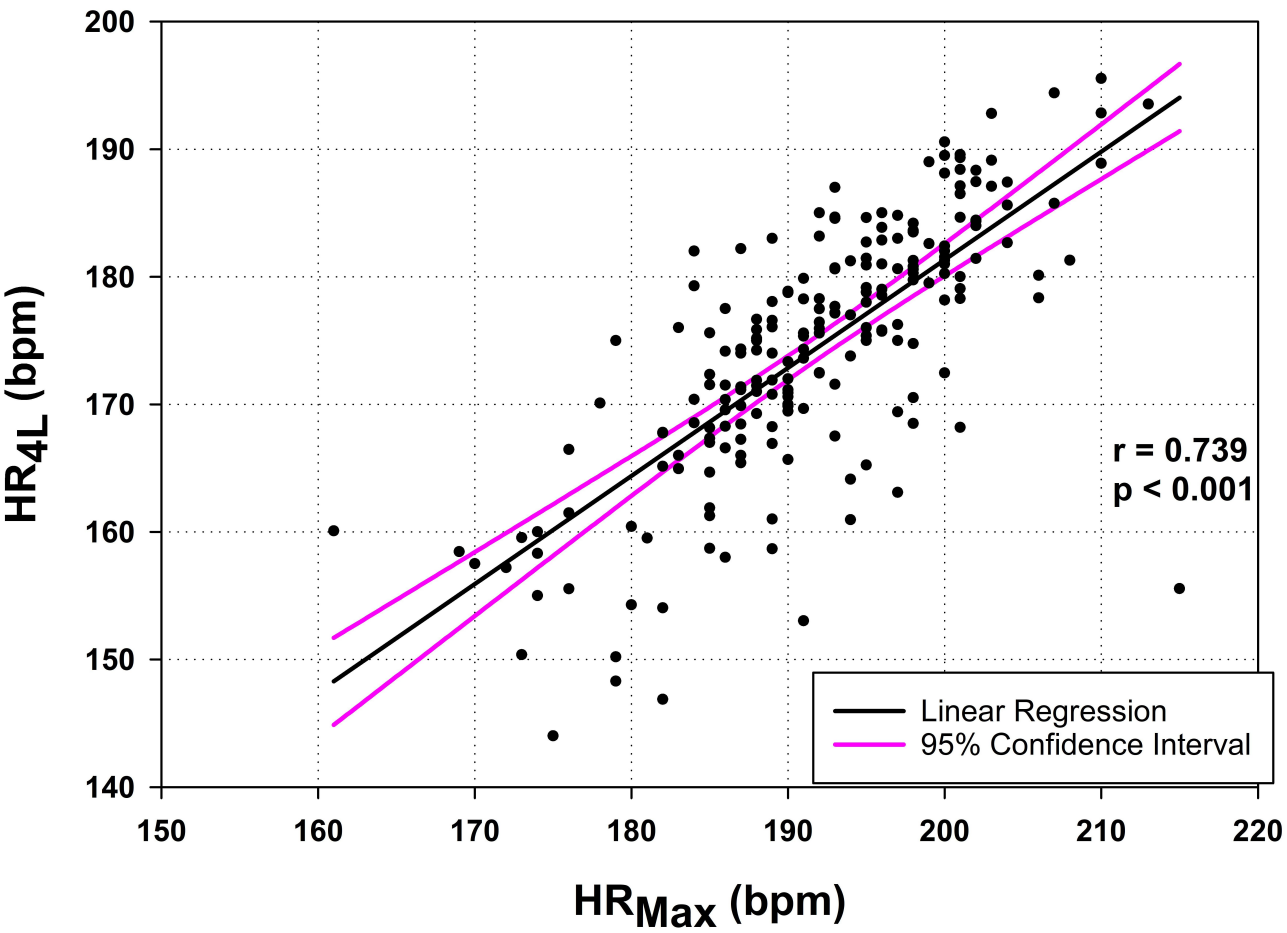
Correlations between treadmill-based heart rate at the 4L threshold (HR_4L_) and maximal heart rate (HR_Max_) in professional adult soccer players (*n* = 206). 95%CI: 0.671-0.796.

**Figure 6:**
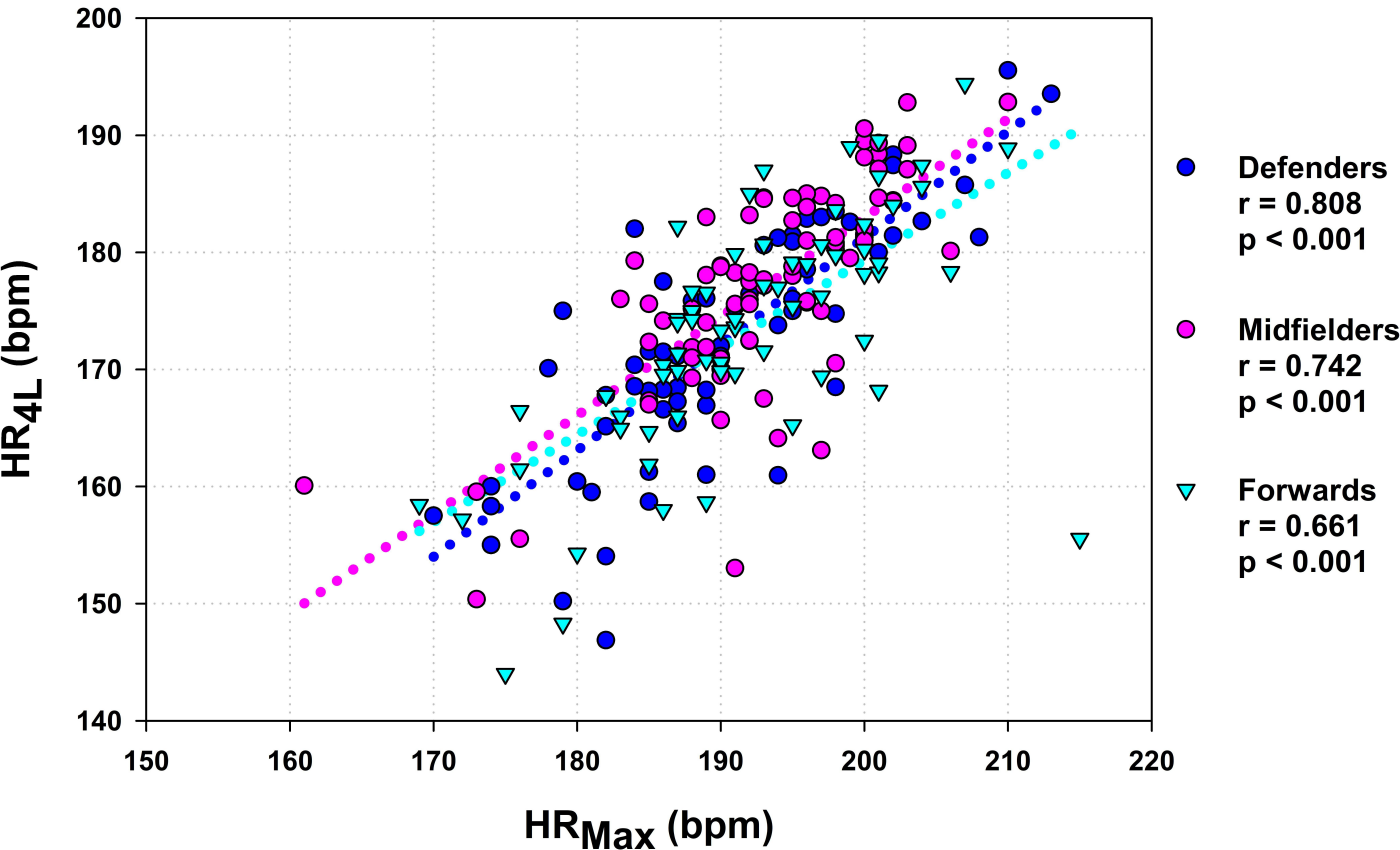
Correlations between treadmill-based heart rate at the 4L threshold (HR_4L_) and maximal heart rate (HR_Max_) in professional adult soccer players in different positions. 95% CI in DEF: 0.704-0.878, in MID: 0.618-0.830, in FW: 0.499-0.778. DEF: defenders; MID: midfielders; FW: forwards.

In sensitivity analyses using the underlying non-normalized variables, the association between V4L and HR_4L_ was weaker (Spearman's *ρ* = 0.284, *p* < 0.001) than that between the corresponding normalized indices (*ρ* = 0.695, *p* < 0.001). V4L was also associated with V_Max_ (*ρ* = 0.613, *p* < 0.001), while HR_4L_ was associated with HR_Max_ (*ρ* = 0.758, *p* < 0.001). In multiple linear regression analysis, V4L remained independently associated with HR4L after accounting for HR_Max_ and HR_Base_ (*β* = 0.408, *p* < 0.001).

### 3.4 Multinomial regression analysis of playing position

In multinomial logistic regression, the model including the submaximal variables V4L and HR4L was significantly associated with playing position (*χ*^2^(4) = 20.335, *p* < 0.001; Nagelkerke R^2^ = 0.106) and correctly classified 46.6% of players. In contrast, the model including VMax and HRMax did not reach statistical significance (*χ*^2^(4) = 8.793, *p* = 0.066; Nagelkerke R^2^ = 0.047), with an overall classification accuracy of 43.2%. The combined model including all four variables was significant (*χ*^2^(8) = 20.826, *p* = 0.008), but showed only a small descriptive increase in Nagelkerke R^2^ (0.108) and classification accuracy (47.1%) compared with the submaximal model.

## 4 Discussion

Individualized profiling of physiological variables, including running capacities, is increasingly important in the characterization of professional level soccer players in different playing positions. There is inconsistency between match-based observational studies, which revealed substantial rise in high-intensity running demands in elite-level soccer (30), and laboratory-based long-term follow-up results, which found no improvement of the maximal aerobic capacity on large number of players over time (16). In accordance with these results, others came to a conclusion, that although high maximal aerobic capacity is a basic requirement in professional level soccer (5, 17), training-related changes of physiological variables, including speed- and heart rate are more detectable at the submaximal anaerobic threshold levels (11, 31, 32), which may provide more sensitive information, than values at maximal exercise performance. Among the treadmill-based markers, speed at the anaerobic lactate threshold was previously reported to characterize professional soccer players (33). Although individualized lactate thresholds are valuable for exercise prescription, the use of a fixed 4L lactate reference (11, 34) provides a standardized physiological benchmark that facilitates longitudinal monitoring and direct comparison between professional players in soccer. The present study demonstrates that standardized treadmill-derived variables obtained at the fixed 4L blood lactate point differentiated playing positions in professional soccer players more consistently than variables at maximal exercise, while remaining strongly associated with maximal performance results. Previous studies have shown, that treadmill-based fitness markers, including maximal- and 4L lactate reference-related running speed, as well as running speed at 80% of the heart rate reserve are strongly associated with match-based markers of external load and internal strain in soccer players (11, 23, 34), although these observations were mainly based on youth players, and on limited number of adult players. The present study expands these observations by demonstrating, in a large cohort of professional adult players, that standardized submaximal treadmill variables provide a consistent physiological characterization of positional differences while maintaining strong associations with maximal exercise responses. Previous match-based reports revealed, that outfield players are required to execute repeated high-intensity efforts, resulting in significant internal load variability and numerous studies have reported that both external and internal load parameters are position-related (6, 35). In our study the most consistent positional differences were observed for treadmill-derived variables obtained at the 4L threshold rather than at maximal exercise, detecting the highest speed at 4L in midfielders, compared to the other outfield player groups, whereas maximal treadmill speed did not differ significantly between playing positions. Importantly, significant positional effects for V_4L_ and HR_4L_ remained after adjustment for age, BMI, club, competitive division, testing phase, and testing year. Playing position remained significantly associated with V_4L_ and HR_4L_, whereas no significant positional effect was observed for V_Max_ or HR_Max_. Thus, the more consistent positional differentiation observed for the submaximal measures was maintained after accounting for these potential confounding factors.

The absolute speed values we found here at 4L were similar to previous studies in soccer players (11, 25). Although previous studies did not find positional differences between speed values at 4L threshold, this controversy can be explained by the different positional classifications and by the lower division level of the players examined previously (25). The superior submaximal running performance of midfielders is physiologically plausible, as these players typically cover the greatest total running distance during matches (36–38) and have previously been reported to exhibit greater aerobic capacity and cardiovascular adaptation than defenders and forwards (16, 39). In youth soccer players, treadmill speed at 4L was previously shown to also have strong correlation with the match-based individual training impulse load values (23) and other studies on adult players at different levels revealed, that treadmill-based maximum speed (34) and the speed at 4L (31) strongly correlate with match-based performance measures. To further improve the physiological interpretation of treadmill performance, we evaluated running speed at the 4L lactate value relative to maximal treadmill speed (V*_4L/Max_* ratio). Expressing submaximal running performance relative to each player's maximal capacity provides a standardized indicator of the aerobic portion of the speed domain, thereby reducing the influence of individual differences in maximal treadmill performance. Similarly to the absolute 4L speed values, V*_4L/Max_* ratio was also found to be the higher in midfielders when compared to the other positions. Moreover, V*_4L/Max_* ratio was inversely associated with La_2min_, the blood lactate concentration measured 2 min after exercise cessation, suggesting that players capable of sustaining a greater proportion of their maximal running speed within the aerobic domain accumulated less lactate during the test. The direction of this association is consistent with a previous report linking superior submaximal endurance performance to lower maximal blood lactate accumulation (31). Heart-rate responses at the fixed 4 mmol·L⁻^1^ reference point showed a complementary positional pattern. In our study, absolute values of the heart rate at 4L were highest in the midfielders, compared to the others, while the positional relation of the maximal heart rate was less consistent. To provide a more individualized representation of internal strain in soccer (20), the heart rates at submaximal effort were expressed as relative to heart rate reserve. Positional distribution showed again highest values in midfielders compared to the other groups for both, absolute heart rate at the 4L threshold and relative HR as percent of HR_Res_ (Table 1). The multinomial regression analyses further showed that the model based on V_4L_ and HR_4L_ was significantly associated with playing position and demonstrated higher pseudo-R^2^ and classification accuracy than the model based on V_Max_ and HR_Max_, which did not reach statistical significance. Adding the maximal variables to the submaximal model resulted in only small descriptive increases in these performance measures. These findings suggest that, within the present cohort, submaximal treadmill responses were more consistently associated with positional differences than the corresponding maximal variables. Correlations between treadmill-based speed and heart rate variables on large number of professional players across different positions may also strengthen the interpretation level of the laboratory tests and support evidence on the performance value of physiological data at submaximal and maximal efforts. Previous report on semi-professional players revealed large correlation between treadmill-based absolute heart rate and speed values at 4L (31). In our study on professional players, although correlations between absolute HR_4L_ and V_4L_ were only moderate and varied between playing positions, expressing heart rate at 4L relative to heart rate reserve (HR*_4L/Res_* ratio) markedly strengthened these relationships. The strong association between HR*_4L/Res_* ratio and V*_4L/Max_* ratio should be interpreted with caution, as both are normalized indices derived from the same incremental test and may therefore be partly affected by mathematical coupling. This interpretation is supported by the weaker association observed between the underlying non-normalized V4L and HR_4L_ variables. However, V_4L_ remained independently associated with HR_4L_ after accounting for HR_Max_ and HR_Base_, indicating that the relationship was not restricted to the normalized indices. The very large correlation between HR*_4L/Res_* ratio and V*_4L/Max_* ratio (Figure 1) indicates that standardized relative markers of internal strain and external load describe closely related physiological characteristics, independently of playing position (Figure 2). These findings support the use of standardized submaximal treadmill-derived variables in professional soccer; however, because both variables are normalized to individual maximal values, the magnitude of this association may be partly influenced by mathematical coupling.

Beyond positional characterization, standardized submaximal treadmill variables remained closely associated with maximal exercise responses. Running speed at the 4L lactate level showed a large correlation with maximal treadmill speed (Figure 3), whereas HR at 4L demonstrated a very large association with maximal HR in the total cohort (Figure 5). These findings indicate that physiologically relevant information obtained during standardized submaximal exercise is strongly related to maximal treadmill performance, despite requiring lower exercise intensity. Similarly, running speed corresponding to 80% of heart rate reserve (V80%HR_Res_) showed a large correlation with maximal treadmill speed, a marker previously reported to correlate with match performance (34). Therefore, our study supports previous observations in semi-professional players, which linked treadmill speed at 80%HR_Res_ with match-related performance (11). Although positional differences in V80%HR_Res_ were less consistent than those observed for 4L-derived variables, its strong association with maximal running performance suggests that heart rate reserve-based treadmill markers may provide useful complementary information during routine laboratory assessment. From a practical perspective, V_4L_ and heart rate-based variables determined at the fixed 4 mmol·L⁻^1^ blood lactate concentration may complement maximal treadmill-derived measures when characterizing the physiological profile of professional soccer players. Relative indices, such as V*_4L/Max_* ratio and HR*_4L/Res_* ratio, may provide complementary information by expressing responses at the fixed 4 mmol·L⁻^1^ reference relative to individual maximal running speed and heart-rate characteristics, respectively. However, these indices require V_Max_ or HR_Max_ as reference values and therefore cannot be derived from a standalone submaximal test without prior or concurrently obtained maximal measurements.

This study has several limitations. The broad positional classification into defenders, midfielders, and forwards may not capture the more specific physiological demands of individual playing roles. Although all participants were professional players, individual familiarity with treadmill running was not formally assessed and may have influenced maximal running performance. Potential club-related differences in training practices and coaching strategies, as well as differences between testing periods, may have influenced physiological responses. Although the adjusted analyses accounted for club, competitive division, testing phase, testing year, age, and BMI, residual confounding related to differences in training practices, coaching strategies, and other unmeasured player characteristics cannot be excluded. The cross-sectional design precludes assessment of physiological changes over time, while the absence of match-derived data limits translation to competitive performance. Only male players were included, limiting generalizability to female players. Respiratory gas exchange was not measured; therefore, the findings characterize running speed, heart rate, and blood lactate responses rather than directly measured maximal aerobic capacity. Although the fixed 4 mmol·L⁻^1^ blood lactate reference improves standardization and comparability, it does not account for inter-individual differences in lactate threshold physiology. Furthermore, the derived relative variables are mathematically dependent on their component measures, including VMax and HRMax, and associations involving these indices may therefore be partly influenced by mathematical coupling. In addition, the stage duration and sampling intervals used in the present protocol may influence the blood lactate–running speed relationship and maximal running speed; therefore, direct comparisons with studies using different incremental protocols should be made cautiously. Testing was partially performed at the beginning of the pre-season, which may not represent peak aerobic fitness despite debated seasonal changes (16, 32, 40, 41). Finally, longitudinal studies are needed to determine the sensitivity of these variables to individual training adaptations and seasonal performance changes.

## 5 Conclusions

This study demonstrates that standardized submaximal treadmill-derived physiological variables provide important complementary information to maximal measures for the physiological characterization of positional differences in professional soccer players. In particular, running speed and heart rate at the fixed 4 mmol·L⁻^1^ blood lactate reference showed positional differences and were associated with maximal running speed and maximal heart-rate response, respectively. Relative indices further characterized these relationships, although their interpretation should consider their dependence on individual maximal reference values. These findings support the combined assessment of submaximal and maximal treadmill-derived variables for physiological profiling of professional soccer players.

## Data Availability

The raw data supporting the conclusions of this article will be made available by the authors, without undue reservation.
